# The reactivity of single magnesium nanoparticles towards corrosion and galvanic replacement

**DOI:** 10.1039/d6nr00806b

**Published:** 2026-06-02

**Authors:** Ambre L. Y. Brabant, Pip J. Knight, Katharine M. Joyce, Mohsen Elabbadi, Vladimir Lomonosov, Christina Boukouvala, Emilie Ringe

**Affiliations:** a Department of Earth Sciences, University of Cambridge Downing Street Cambridge CB2 3EQ UK er407@cam.ac.uk; b Department of Materials Science and Metallurgy, University of Cambridge 27 Charles Babbage Road Cambridge CB3 0FS UK

## Abstract

Magnesium (Mg) nanoparticles are promising for plasmonic applications due to their wide resonance range, biocompatibility, and low cost. The low reduction potential of Mg leads to high reactivity, a double-edged sword yielding fast corrosion in water but also opportunities for synthetic strategies based on galvanic replacement. This study uses single particle dark field scattering to monitor the real-time dynamics of Mg nanoparticle corrosion and galvanic replacement by Pd, Cu, Pt, and Au. We find that while corrosion starts immediately and proceeds gradually, galvanic replacement typically exhibits a significant induction stage, lasting up to two hours, followed by a rapid reaction phase. Results indicate that the induction stage is likely governed by the hydration and breakdown of the protective, native MgO surface. Consistent with this explanation, the duration of the induction stage decreases with increasing precursor concentration, decreasing pH of the metal precursor, and with the addition of water or NaCl known to accelerate MgO hydration. These mechanistic insights provide a foundation for designing the synthesis of Mg-based bimetallic nanostructures for plasmonic applications, as demonstrated for the Mg–Cu system in this paper.

## Abstract 2

The corrosion and galvanic replacement of Mg nanoparticles was studied at the single particle level. The increase in rate of replacement in the presence of few % water in alcohol enables the design of synthetic strategies for Mg-based bimetallics.

## Graphical abstract

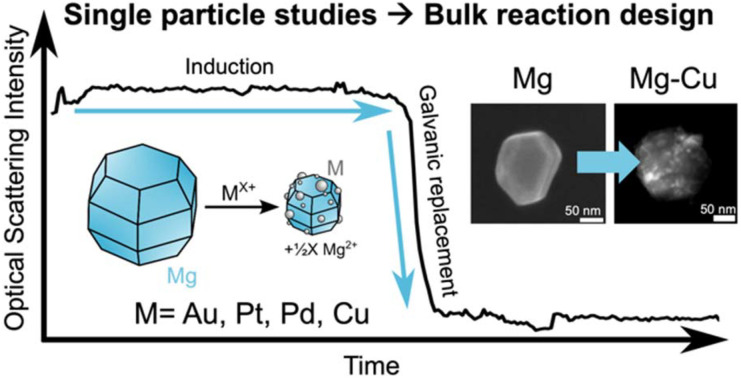

## Introduction

Localised surface plasmon resonances (LSPRs) are light-driven coherent oscillations of conduction electrons in metallic nanoparticles (NPs) that lead to strong, wavelength-dependent optical absorption, scattering, and enhancement of the local electric field. The understanding and control of LSPRs has flourished over the past decades, paving the way for multiple established and potential applications of plasmonic structures, ranging from biosensing to light-enhanced reactions and cancer ablation. A challenge in the engineering of plasmonic-powered effects is the limited number of materials capable of sustaining LSPRs at visible and near-infrared wavelengths, useful for solar harvesting and *in vivo* applications, respectively.

Mg is a plasmonic metal attracting growing interest.^1–8^ Indeed, Mg NPs can sustain LSPRs across the ultraviolet, visible, and near-infrared range, with a plasmonic quality factor better than Al's in the 300–900 nm range, and better than Cu and Au at wavelengths below 500 nm.^9^ Mg is also earth-abundant (8^th^ most abundant element in the crust) and biocompatible (Mg^2+^ is an essential nutrient). However, persistent concerns about Mg NPs include their safety and reactivity. The reactivity of Mg is rooted in its low reduction potential of −2.37 V *vs.* standard hydrogen electrode.^10^ Mg reacts with O_2_, even at very low partial pressures, to form MgO, a stable, self-limiting surface oxide.^11^ While the stability of plasmonic Mg NPs protected with native MgO in air has been demonstrated up to 400 °C and in moist air between 200–400 °C,^11^ their reaction in water is rapid and leads to “transient”, *i.e.*, erasable, structures.^7^

Mg's reactivity and thermodynamically stable species in water are potential and pH-dependent, as described in its Pourbaix diagram.^12^ Without an applied potential, Mg corrodes to Mg^2+^ ions below pH 11. The low reduction potential comes with advantages, too, as galvanic replacement of Mg is thermodynamically favourable for most d-block metals, and this reaction has been used as a synthetic strategy to produce decorated structures,^13–17^ including Mg–Pd and Mg–Au architectures used in plasmon-enhanced catalysis.^16–18^

While the outcome, at the bulk level, of surface oxidation, corrosion, and galvanic replacement of Mg NPs is increasingly well understood, recent advances in nanoscience indicate that bulk observations and single particle reactivity can be strikingly different, the latter offering real reaction mechanism insights. For instance, Smith *et al.* demonstrated that galvanic replacement of Ag by Au is much faster at the single particle level than when viewed as an ensemble and exposed the importance of pit formation in the replacement process.^19^ Further, Sterl *et al.* showed the importance of individual crystals in the dynamics of hydrogen absorption in polycrystalline Mg disks.^20^

Here, we interrogate the time-resolved, single particle level behaviour of Mg corrosion and galvanic replacement by a variety of metals. The changes in optical scattering from plasmonic Mg NPs is first numerically modelled to understand the expected size dependence. Then, the time-resolved scattering response of a large number of single particles is used as a proxy for reaction progress, leading to the observation of reaction rates correlating with water concentration. Next, this approach is applied to galvanic replacement of Mg with Pd, Cu, Au, and Pt, highlighting not only rapid reactions at the single particle level, but also the effect of corrosion-enhancing co-agents including water and NaCl. Lastly, we implement the insights from single particle studies to design an experiment for the bulk galvanic replacement of Mg by Cu. Together, these results reveal the dynamics of corrosion and galvanic replacement of plasmonic Mg NPs, providing a framework to design synthetic strategies for bimetallic structures.

## Materials and methods

### Nanoparticle synthesis

Platelet Mg NPs, hexagonal in shape, were synthesised by a procedure described in Wayman *et al.*,^21^*i.e.*, by the reduction of MgBu_2_ by lithium naphthalenide in THF in the presence of poly(vinylpyrrolidone) *M*_w_ = 10 000. Faceted spheroidal Mg NPs were synthesised by a seed-mediated reduction procedure described in Lomonosov *et al.*^22^ Size distributions obtained from secondary electron microscopy (SEM) images are reported in Fig. S1. The average length (longest axis as measured in images) of the faceted spheroidal NPs was 132 nm (standard deviation 20 nm), with an average aspect ratio of 1.2, while the average length of the platelet Mg NPs (longest axis) was 520 nm (standard deviation 232 nm), with an average aspect ratio of 2.0.

### Dark field optical microscopy

**Fig. 1:**
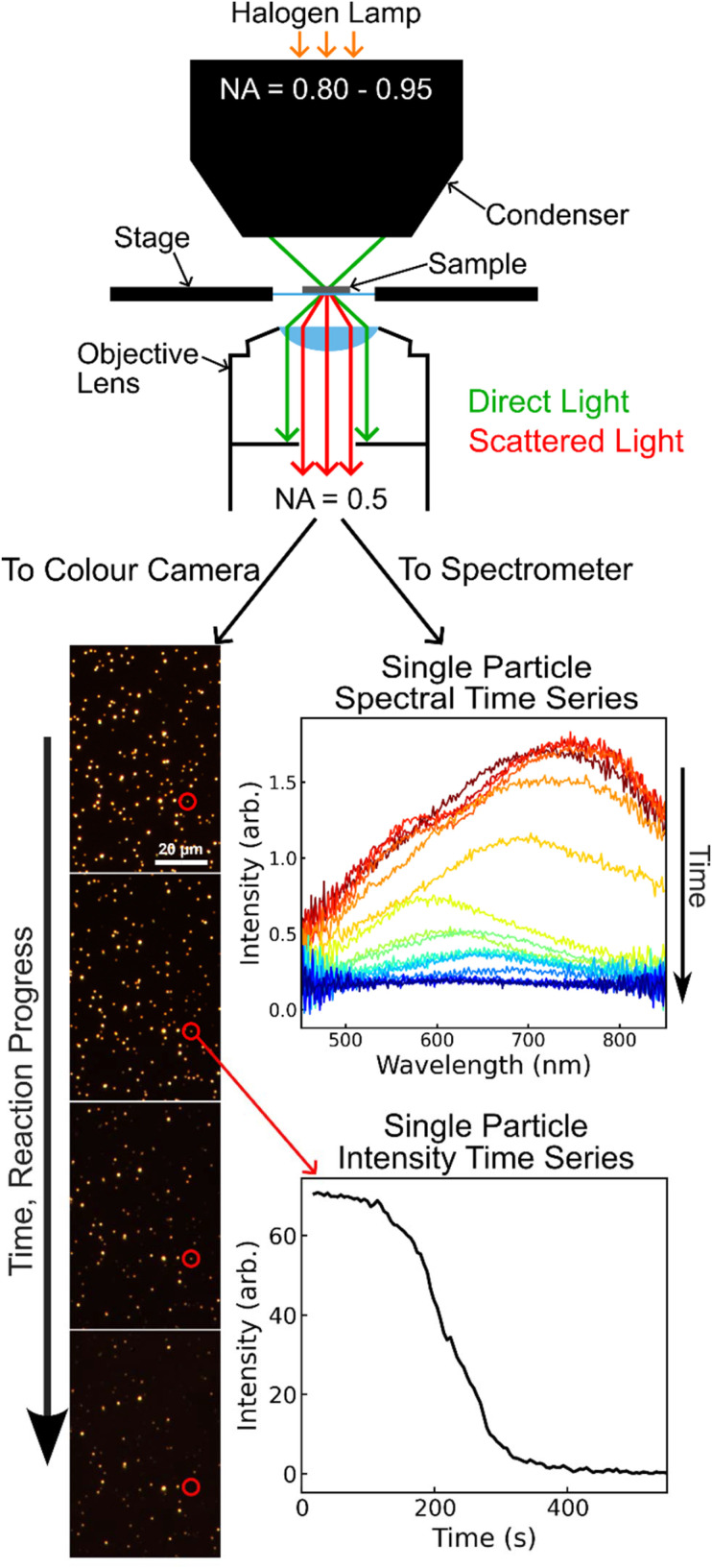
Single particle dark field optical scattering. A hollow cone of white light is directed to a drop-cast Mg NP sample, leading to scattering collected in imaging (camera) or spectroscopy (spectrometer and CCD) mode. Time series of the total scattering intensity are extracted in imaging mode, while time series of the scattering spectrum are extracted in spectroscopy mode.

Reactions of Mg NPs with water and metal salts were studied *in situ* in a dark field optical setup (Fig. 1) in a Nikon Eclipse Ti2 inverted optical microscope as described in Kumar *et al.*^23^ White light from a Nikon D-LH/LC 12 V 100 W halogen lamp was directed through a Nikon dark field condenser with numerical aperture (NA) 0.80–0.95. A Nikon CFI Plan Fluor 100XS oil immersion objective lens with NA set to 0.5 collected the light scattered by the sample, which was directed either to a Thorlabs CS505CU Kiralux 5.0 MP Color CMOS Camera or a Princeton Instruments IsoPlane SCT320 spectrometer fitted with a 50 g mm^−1^ grating followed by a Princeton Instruments ProEM HS 1024 × 1024 EMCCD (Na_2_PdCl_4_, HAuCl_4_, and Na_2_PtCl_4_ reactions) or a Princeton Instruments PIXIS 256E CCD (H_2_O and CuCl_2_ reactions).

In each experiment, a glass cover slip was rinsed with isopropanol before drop-casting 4–10 µL of a 0.3–0.6 mM suspension of NPs in isopropanol. The Mg NPs were then put into contact with a liquid reagent inside the microscope using one of three setups shown in Fig. S2. In the drop setup, 50 µL of reagent was pipetted onto the slide. In the open-cell setup, a piece of 2.5 mm thick silicone with a 1 cm × 1 cm square hole was placed on the cover slip and 250 µL of reagent was pipetted into the hole. In the closed-cell setup, the top surface of the cell was sealed with a second glass cover slip. The drop setup was used for reactions taking seconds to minutes and the closed-cell setup for those lasting >10 minutes to minimize the impact of solvent evaporation. The open-cell setup was used for reactions in ethanol, taking seconds to minutes, for which a drop setup was not appropriate due to rapid evaporation in the illuminated regions.

### Single particle reactions

#### Water

Faceted spheroidal Mg NPs were exposed to mixtures of isopropanol and deionised water, with water concentration varying from 10 vol% to 60 vol%. Experiments were carried out in the drop setup (except 10 vol%, closed-cell setup) and recorded with the colour camera at a rate of 0.2–1 frames per second (fps).

#### Palladium

Platelet Mg NPs were exposed to solutions of Na_2_PdCl_4_ (99.99%, Sigma-Aldrich) in isopropanol ranging from 0.5 mM to 2.0 mM. Experiments were also carried out with 0.5 mM Na_2_PdCl_4_ + 2 mM NaCl (99.95%, Fisher Chemical), and with 1.5 mM Na_2_PdCl_4_ + 1 vol% water. Experiments were carried out in the closed-cell setup and recorded with the colour camera at 0.025–0.1 fps. Additional reactions with 1.0 mM Na_2_PdCl_4_, reported in the SI, were recorded with the spectrometer every 4 min.

#### Copper

Faceted spheroidal Mg NPs were exposed to CuCl_2_ (99%, Sigma-Aldrich) in ethanol at concentrations between 0.1 mM and 7.4 mM; ethanol was chosen as the solvent for CuCl_2_ to allow for a large concentration range. Experiments were carried out using the open-cell setup; they were recorded with the colour camera at 0.2–1.0 fps and, for 0.1 mM CuCl_2_, in spectroscopy mode every 2.3 s.

#### Gold

Platelet Mg NPs were exposed to 0.05 mM HAuCl_4_ (99.999%, Sigma-Aldrich) in isopropanol in the closed-cell setup and recorded with the colour camera at 0.5 fps.

#### Platinum

Platelet Mg NPs were exposed to 1.0 mM Na_2_PtCl_4_ (99.95%, Alfa Aesar) in isopropanol in the closed-cell setup and recorded with the colour camera at 0.5 fps.

In all reactions, reagents were in excess of Mg. Water was in excess by a factor of >10^4^; metal salts were in excess by factors ranging from approximately 3 to 300.

In a given experiment, 16–44% of intensity-time series with Na_2_PdCl_4_ contained more than one step and 17–56% for CuCl_2_ (Table S1); such series may correspond to small aggregates in which different particles react at different times. These time series were not analysed for kinetics and are available upon request from the authors.

### Colloidal galvanic replacement reactions

Colloidal faceted spheroidal Mg NPs (∼2 mM) were exposed to 1.7 mM CuCl_2_ in anhydrous ethanol (and just anhydrous ethanol for control experiments). Deionised water was added in concentrations of 0, 1.7, 3.3, and 8.3 vol%, corresponding to 0, 0.1, 0.2, and 0.5 mL in the total 6 mL reaction volume. Reactions were stirred at room temperature for 10 minutes after mixing. Following this, reactants were removed by two cycles of centrifugation (10 min at 800 RPM) and redispersion in anhydrous ethanol and one cycle of centrifugation and redispersion in anhydrous isopropanol. NPs were redispersed in isopropanol, transferred to a PMMA cuvette, and UV-Visible extinction profiles were obtained with a Thermo-Scientific Evolution 220 spectrophotometer.

### Data processing – imaging

Raw data from the colour camera consisted of stacks of images taken at regular time intervals and was processed using ImageJ. Firstly, a SIFT registration algorithm^24^ built into ImageJ was used to correct for image drift if needed. Thresholding and automatic particle detection were then applied to extract the scattering intensity-time series of individual particles, with the intensity being averaged over the same particle area through the time series to ensure consistency. Particle intensity-time series were processed in Python including background corrections and automatic spike detection and removal.

In addition to the above processing, in the experiments with CuCl_2_ the particles which disappeared in the time gap between adding the reagent and starting the recording (∼10–30 s) were counted by comparing the first frame of the camera recording with an image taken before adding the reagent. Linear models were fit by least-squares regression to single particle intensity-time series from the experiments with CuCl_2_, in the interval between 80% and 20% of the initial intensity (Fig. 6). However, in a subset of cases for 0.1 mM CuCl_2_, a line was fit down to only 50% if the time series diverged from linearity by sloping off to shallower gradients. Within the same reaction, the number of particles from which a reaction gradient was obtained can be lower than the number of NPs for which an induction stage duration was measured, owing to particles decaying before the recording started.

### Data processing – spectral time series

Spectra were background subtracted and divided by the lamp output profile (Fig. S3) as described in Kumar *et al.*^23^ The location of a particle in the slit was identified and averaged over three pixels along the spatial dimension to reduce noise. This gave the particle scattering intensity as a function of wavelength and time.

### Electron microscopy

NPs were imaged from drop-cast solutions on indium tin oxide (ITO)-coated coverslips using a Nova NanoSEM L50 field emission gun SEM operated at 15 kV. All SEM images shown in the main manuscript were acquired with an Everhart–Thornley secondary electron detector. Additionally, the instrument was equipped with a concentric-backscattered detector and an Oxford Instruments energy dispersive X-ray spectrometer (EDS); results from these detectors are reported in the SI. SEM was also used to characterise the size distributions of Mg NPs used in the reactions (Fig. S1). High-angle annular dark field scanning transmission electron microscopy (HAADF-STEM) images were acquired on a FEI Osiris operated at 200 kV, with NPs drop-cast on carbon-coated Cu grids.

### Numerical simulations

**Fig. 2:**
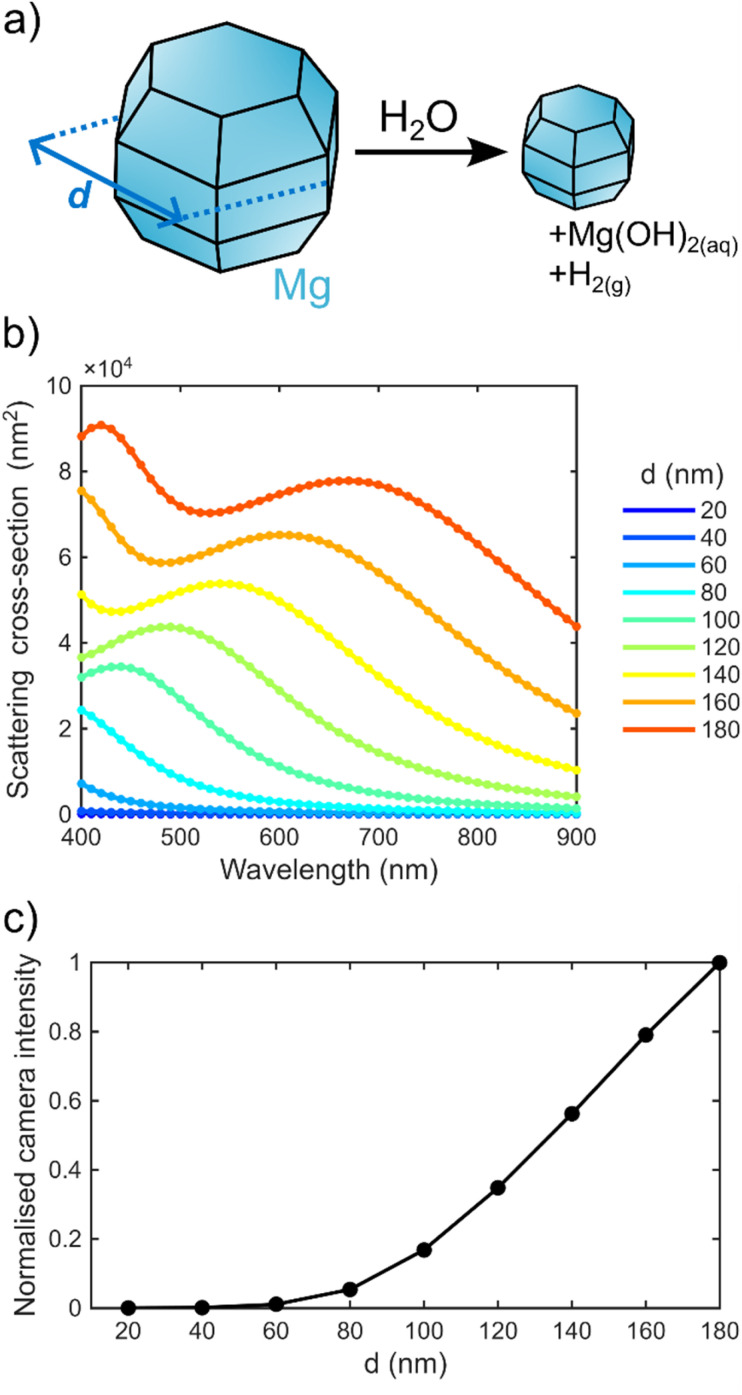
Numerical simulations of Mg NPs scattering. (a) Schematic of the reaction of Mg NPs with water, showing the shape used to approximate the faceted spheroidal Mg NPs and the definition of the tip-to-tip distance *d*. (b) Scattering cross-section as a function of *d*, for a Mg NP on a glass substrate in isopropanol. (c) Calculated intensity in the colour camera as a function of *d*. Markers in (b) and (c) indicate calculated points.

Scattering cross-sections for the faceted spheroidal NPs were calculated numerically with DDSCAT, a Maxwell solver using the discrete dipole approximation,^25^ with an inter-dipole distance of 0.7–3.0 nm depending on the NP size. A Wulff construction using Crystal Creator^26^ with surface energies from Kopač Lautar *et al.*^27^ (Table S2) was used to approximate the shape of the faceted spheroidal NPs. A cuboidal glass substrate of thickness 40 nm and side length 80 nm + the tip-to-tip width of the faceted spheroidal NPs (*d* in Fig. 2) was added to account for the effect of the glass cover slip. The dielectric function of Mg was taken from Palik,^28^ the ambient refractive index was set to 1.38 (isopropanol), and the refractive index of the glass, to 1.52. Light was incident at 31° to the substrate and polarised in a vertical plane; this reproduced the inclination of the light cone output by the dark field condenser and was a good approximation for unpolarised light given the 1.2 aspect ratio of the faceted spheroidal NPs. Scattering cross-sections (*σ*) were calculated for wavelengths (*λ*) between 400 nm and 900 nm in 10 nm steps. The expected relative intensity in the colour camera was then calculated as follows:1
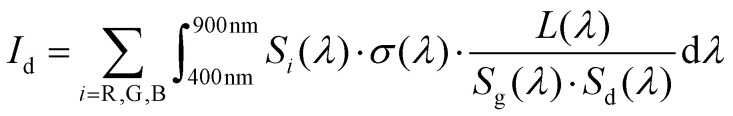
where *I*_d_ is the relative intensity measured by the camera, *L* is the lamp spectrum measured with the spectrometer (Fig. S3), and *S*_d_, *S*_g_, *S*_*i*_ are the spectral responses of the CCD, spectrometer grating, and *i*^th^ channel of the colour camera respectively (spectral responses provided by Thorlabs and Princeton Instruments). The integrand was numerically integrated over wavelength and summed over the three channels of the colour camera.

A surface film consisting of MgO and/or Mg(OH)_2_ is expected to be present on the NPs at all times; studies of the reaction of Mg with water show variability in the structure, thickness, and composition of this film, suggesting its sensitivity to experimental conditions.^29–39^ Because of the uncertainty in the characteristics of the surface film, for simplicity the NPs were modelled without oxides, and considering the trends rather than aiming for a quantitative match. We do not expect this approximation to significantly affect the derived conclusions, because previous numerical results show that the presence of an MgO shell shifts LSPRs to longer wavelengths with little effect on the LSPR intensity.^40,41^

## Results and discussion

### Reactivity in water

We first explore numerically the changes in optical scattering of a Mg NP as its size decreases, mimicking a reaction with water. Smaller NPs are expected to produce a lower LSPR intensity and higher resonance energy.^42,43^ Simulations of the dark field scattering spectrum of faceted spheroidal Mg NPs with various diameters *d* (Fig. 2a), on a 1.52 refractive index (RI) glass substrate and immersed in an isopropanol medium (RI = 1.38) were obtained using the discrete dipole approximation.^25^ Results confirm the expected intensity and wavelength shifts (Fig. 2b), while the total intensity expected experimentally, corrected for the wavelength-dependent sensitivity of the optical setup, decreases monotonically with NP size (Fig. 2c).

**Fig. 3:**
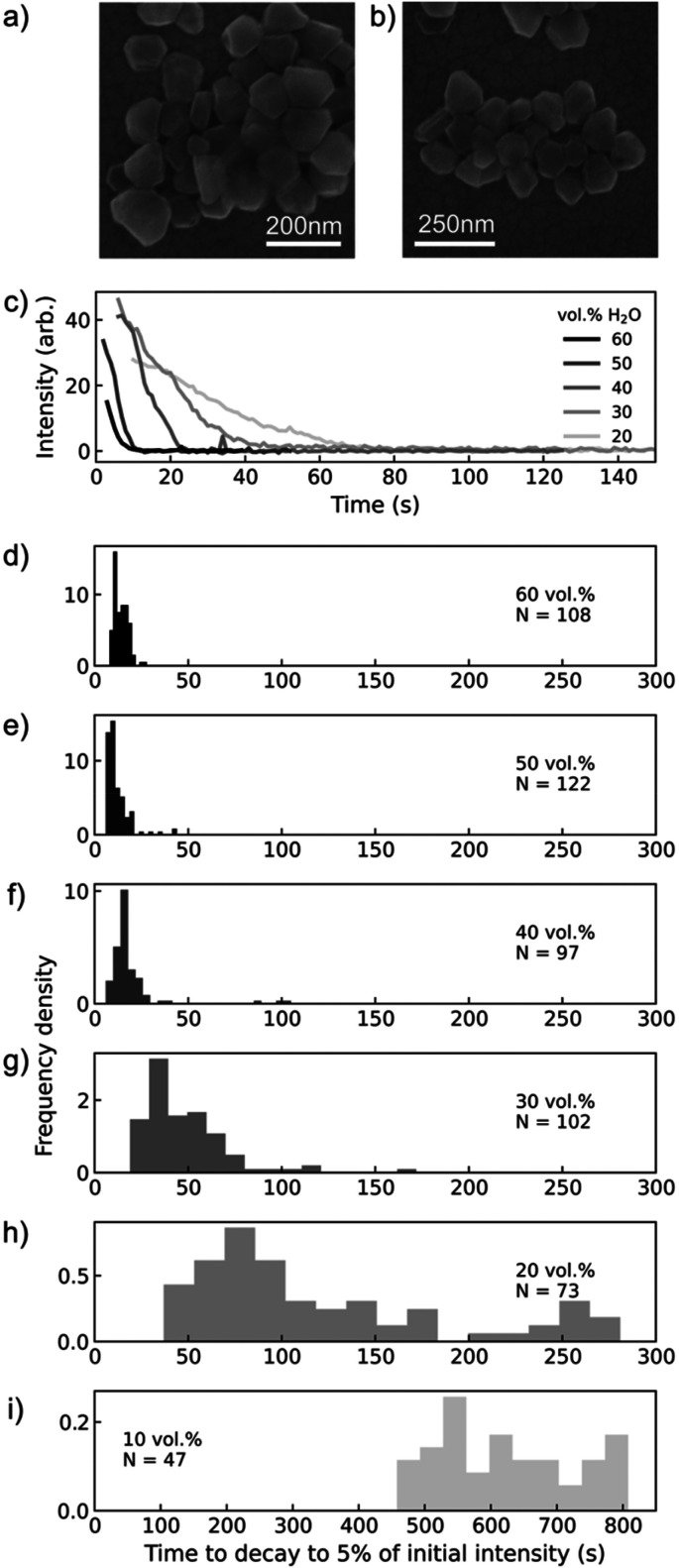
Corrosion of faceted spheroidal Mg NPs by water–isopropanol mixtures. (a and b) SEM images of faceted spheroidal NPs before water exposure, for a concentrated suspension. (c) Single particle scattering as a function of time for representative NPs from experiments at five different water concentrations. (d–i) Distributions of decay time, defined as the time for the intensity to decay to 5% of its initial value, for different water concentrations. *N* is the number of particles in each distribution. Note that the time axis in (i) is larger than in the other plots.

To probe corrosion experimentally, Mg NPs were synthesised using a previously reported colloidal reduction procedure, yielding faceted spheroidal NPs (Fig. 3a and b) with an average side length of 132 nm (standard deviation 20 nm, Fig. S1). Bulk corrosion experiments were first performed (Fig. S4), showing the decrease in LSPR intensity of Mg NPs over time in 5 vol% water in isopropanol. This ensemble-averaged data is difficult to interpret, however, because water-induced aggregation cannot be separated from the effects of the reaction itself, and neither can the signal from single NPs and that of aggregates. Therefore, we turned to single particle studies, implemented in a dark field optical scattering microscope (Fig. 1) equipped with a spectrometer/CCD for spectral acquisition as well as a colour camera for rapid, large-scale scattering intensity monitoring.

Time series of the scattering intensity of single faceted spheroidal NPs during reaction with water–isopropanol mixtures were approximately linear (Fig. 3c), as evidenced by high *R*-squared values (Fig. S5). Apparent reaction completion was reached between 20 s and 800 s for water concentrations between 10 vol% and 60 vol%, with the distribution of reaction times narrowing and shifting to lower mean times as the water concentration increased (Fig. 3d–i). This observation is not an artefact of variable starting NP size distributions, as the same samples were used; as expected there was no statistically significant difference between the distributions of single particle scattering intensity (a proxy for size) at the start of the experiments with different water concentrations (Fig. S6), nor any trends for the scattering intensity across the different experiments.

### Tracking galvanic replacement reactions

#### Overview

**Fig. 4:**
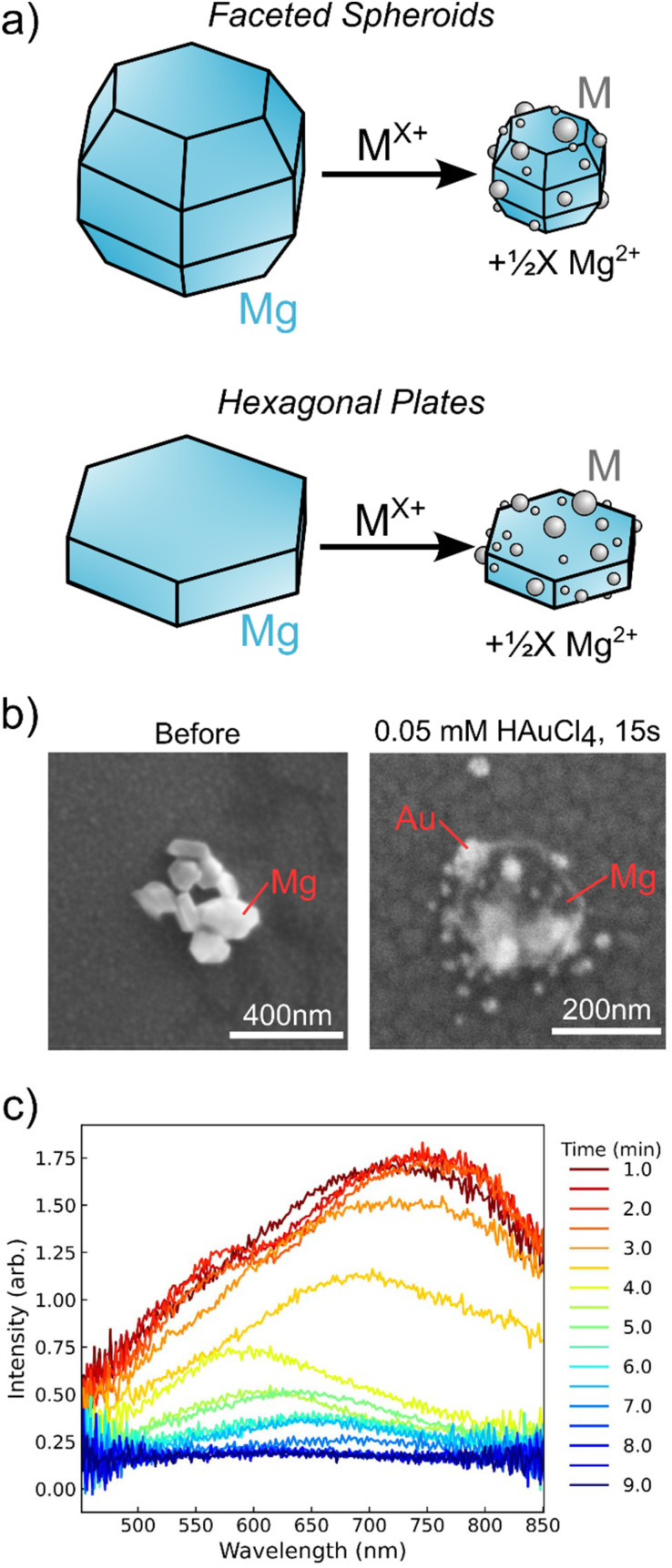
Galvanic replacement reactions. (a) Schematic representation of the galvanic replacement reaction between a Mg NP and a metal salt. (b) SEM images of Mg NPs to which no reagent has been added, and a Mg NP that has reacted with 0.05 mM HAuCl_4_ for 15 seconds, displaying metallic Au decorations. (c) Experimental scattering spectrum of a single faceted spheroidal Mg NP as a function of time during reaction with 0.1 mM CuCl_2_.

Similarly to the tracking of Mg corrosion described above, single particle approaches also offer opportunities to explore the dynamics of galvanic replacement of Mg NPs by ions with more positive reduction potentials (most metals except the alkali).^44–46^ Here, we further our exploration of single particle reactivity by investigating the galvanic replacement of Mg, shown schematically in Fig. 4a. Briefly, Mg NPs were drop-cast and exposed to various concentrations of the metal ions in solution. We first investigate and compare the behaviour of the noble metals Pd, Au, and Pt using platelet Mg NPs. Then, we use spheroidal Mg NPs discussed above to delve in-depth in the understanding, then design, of galvanic replacement reactions by Cu.

SEM images (Fig. 4b and S7–S9) and SEM-EDS spectra (Fig. S7) of the structures produced by partial reactions (quenched after variable reaction times by rinsing with isopropanol) indicate that the contrast of the Mg core, a proxy for thickness, decreases as Mg is oxidised to Mg^2+^, while the replacing metal (initially an oxidised salt in solution) is reduced and deposited as a solid on the surface of the Mg core. The morphology of the deposited metal, discrete islands rather than continuous layers, is consistent with previous results for bulk GR studies.^44^ Note that there is no miscibility between the Mg and the replacing metals investigated here (Pd, Pt, Au, Cu),^47–50^ so that alloying between the replaced and replacing metals is unlikely to play a role, unlike in the galvanic replacement of Ag by Au.^19^

The appearance of Pd islands in the galvanic replacement of platelet Mg NPs by Na_2_PdCl_4_ is time-dependent, with no Pd structures observed in the first 15 min of the reactions with 0.5 mM and 1.0 mM Na_2_PdCl_4_. Pd appears earlier at higher concentrations, visible after 7.5 minutes at 1.5 mM (Fig. S8). While instructive of the morphology of replaced structures, such quenched experiments provide little insight into the reaction kinetics.

By consuming the Mg core and depositing another metal, galvanic replacement is expected to affect the optical scattering of the NPs, providing means to track reaction progress in real time. Experimentally, the LSPR scattering intensities decreased as reactions proceeded, while resonance wavelengths shifted in variable directions (Fig. 4c for Cu and Fig. S10 for Cu and Pd). Upon reaction completion, the scattering signal essentially vanished (Fig. 4c for Cu, Fig. S10 for Cu and Pd, and Fig. S11 for Au, Pt, and Pd), even when the replacing metal itself was plasmonic (*e.g.*, Cu and Au). This is likely due to Cu oxidation and the production of Au nanostructures too small to significantly scatter.^42^

Since the addition of small metal decorations does not significantly contribute to the experimentally observed optical response, the modelled size dependence of the scattering of a faceted spheroidal Mg NP (Fig. 2) still applies: the single particle intensity in the camera decreases monotonically with NP size. This also applies to platelet Mg NPs (Fig. S12).

#### Galvanic replacement by Pd

The partial reaction of platelet Mg NPs with Na_2_PdCl_4_ produced Mg cores decorated by Pd NPs (Fig. S7 and S8) as previously reported by Lomonosov *et al.*^16^ The products of complete galvanic replacement displayed only Pd, sometimes loosely attached to small fragments of oxidized Mg (Fig. S8).

**Fig. 5:**
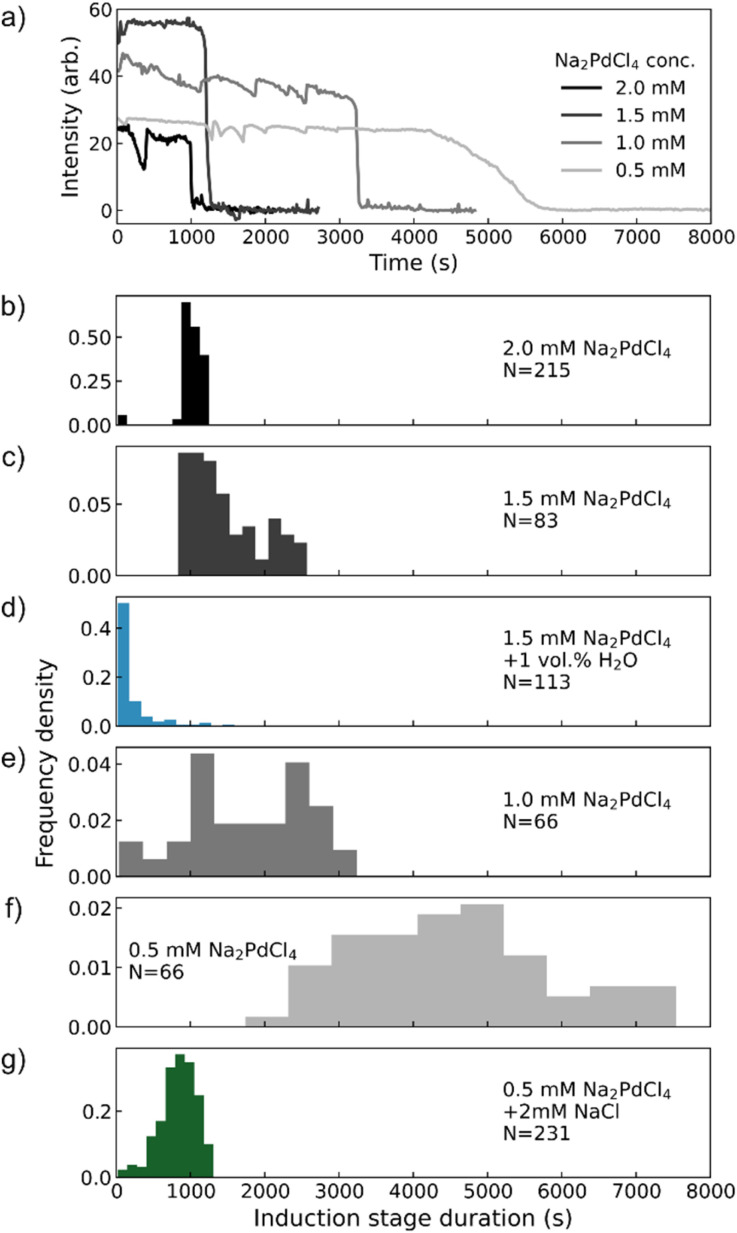
Galvanic replacement of platelet Mg NPs with Na_2_PdCl_4_. (a) Single particle scattering intensity as a function of time for representative NPs from experiments at four different concentrations. Defocussing and refocussing in the microscope led to dip-like features observed concurrently for all particles within an experiment. (b–g) Distributions of induction stage duration, defined as the time for the intensity to decay to 60% of its initial value.

Intensity-time series of single platelet Mg NPs reacting with Na_2_PdCl_4_ consisted of two distinct stages (Fig. 5a). The induction stage, lasting up to two hours where little to no change in intensity was observed, preceded a sharp decrease to noise-level scattering intensity (the reaction stage) for Na_2_PdCl_4_ concentrations between 0.5 mM and 2.0 mM. This behaviour starkly contrasts with the gradual and linear decrease in scattering intensity during the corrosion reaction and is very similar to the Ag replacement by Au observed by Smith *et al.*^19^ Spectrally resolved time series also confirm the presence of induction, showing the overall scattering profile staying constant before undergoing a sharp overall intensity drop (Fig. S10).

The distribution of induction stage durations shifted to shorter mean times and narrowed with increasing Na_2_PdCl_4_ concentration (Fig. 5b, c, e and f), similarly to the replacement of Ag by Au.^19^ The reaction was very fast compared to the sampling needed to measure the induction stage (in all experiments except at 0.5 mM Na_2_PdCl_4_, up to 82% of reactions completed within 20 s) so that a rate could not reliably be extracted for the reaction stage.

Smith *et al.*^19^ attributed the induction stage to the time needed to form a void of a critically stable size in the Ag NP undergoing galvanic replacement. Here, the induction stage could similarly represent the time taken to form a critically stable nucleus of Pd metal on the surface of the Mg core, after which the reaction can proceed rapidly. Despite this explanation being consistent with the shortening of the induction stage with increased concentrations of Na_2_PdCl_4_, it cannot account for extremely long induction times (up to 2 h). Indeed, due to the large difference in the reduction potentials of Mg and Pd, the characteristic time of Pd nucleus formation is expected to be moderately short.

To describe the oxidative action of hydrogen peroxide on Ag NPs, Naik *et al.*^51^ described a “sawing” mechanism, where Ag NPs are broken up into multiple smaller NPs. This mechanism is unlikely here: the smooth nature of single particle intensity-time series decay suggests progressive dissolution of Mg NPs rather than multiple sawing events.

Alternative to these explanations, the prolonged induction behaviour can be attributed to mass transport limitations observed at the initial stages of galvanic replacement. Due to high surface reactivity, as-synthesised Mg NPs are always covered with a thin (5–20 nm) native self-limiting oxide layer, which protects the NPs from further oxidation.^11^ The oxide layer is expected to substantially hinder galvanic replacement by acting as a mass-transport barrier, leading to the long induction times required for Pd ions to reach the Mg core. Consistent with this hypothesis, adding 1 vol% water to the reagent mixture reduced the induction stage duration from 15–40 min to <5 min in most cases (Fig. 5c*vs.*5d), by converting MgO into less passivating Mg(OH)_2_.^29,46^ Control experiments with 1 vol% water in isopropanol and no Na_2_PdCl_4_ (Fig. S13) show only a 40% loss in scattering intensity over 30 minutes, demonstrating that this acceleration of the induction stage does not simply result from direct oxidation of the Mg by water. Furthermore, when 2 mM NaCl was present during galvanic replacement of Mg NPs with 0.5 mM Na_2_PdCl_4_, the induction stage duration decreased from 30–125 min to <20 min (Fig. 5f*vs.*5g). Given Cl^−^ ions can accelerate the hydration of MgO,^38,39,52–54^ the observed shortening of the induction stage by the addition of NaCl or the increase in Na_2_PdCl_4_ concentration (also affecting Cl^−^ concentration) is consistent with the hydration hypothesis.

The hydration of the MgO film should also be a relevant process in the reaction with water–isopropanol mixtures, in which, however, no induction stage is observed. The high concentration of water probably meant the hydration of MgO was too fast to appreciably affect the scattering intensity-time series or to produce an induction stage.

There is no correlation between single particle initial intensity and induction stage duration (Fig. S14). Given the expected relationship between NP size and scattering intensity, this observation suggests the induction stage duration is independent of the starting Mg NP size, consistent with a mechanism involving the hydration of the (size-independent) MgO layer.

Similarly to the reaction with Pd, the partial reaction of platelet Mg NPs with HAuCl_4_ and Na_2_PtCl_4_ produced Mg cores decorated with Au or Pt islands, while the complete reaction produced NPs consisting only of the replacing metal (Fig. S9 and S15), as confirmed by elemental analysis with SEM-EDS (Fig. S7). The replacement of Mg by Pt displayed an induction stage of 100–150 s for 1.0 mM Na_2_PtCl_4_, with a narrow distribution of decay times (Fig. S15). Meanwhile, for the replacement of Mg by Au, the induction stage, if present, was shorter than the experimental starting time (t*) of 30 s, and the reaction time was fast (50–250 s) considering the comparatively very low 0.05 mM HAuCl_4_ concentration (Fig. S15). The timescale of galvanic replacement for these metal salts thus goes, from slowest to fastest, as Na_2_PdCl_4_ > Na_2_PtCl_4_ ≫ HAuCl_4_. The marked difference for the reactivity of Au is likely due to the formation of an acidic solution known to facilitate MgO hydration.^55^

#### Galvanic replacement by Cu

The galvanic replacement of Mg by Cu was performed for spheroidal Mg NPs; their narrow size distribution and shape homogeneity^22^ allows the mitigation of potential size and shape effects on single particle measurements. Similarly to the replacement of Mg by Pd, Pt, and Au, the partial reaction of Mg NPs with CuCl_2_ produced Mg cores decorated by a Cu-dominated solid as evidenced by SEM-EDS (Fig. S7). These decorations are likely an oxidised form of Cu resulting from water and air exposure during and/or after reaction. However, the absence of Cl in the EDS spectrum rules out precipitation of CuCl_2_.

**Fig. 6:**
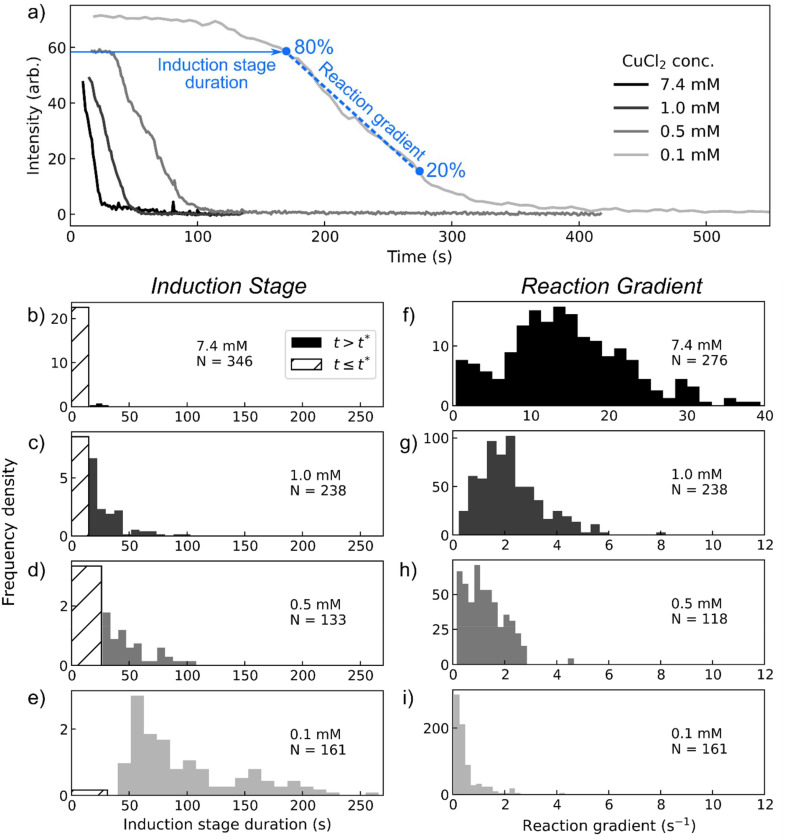
Galvanic replacement of faceted spheroidal Mg NPs with CuCl_2_. (a) Scattering intensity as a function of time for representative NPs from experiments at four different concentrations of CuCl_2_. Blue annotations define induction stage duration (time for the intensity to decay to 80% of its initial value) and reaction gradient (best-fit linear gradient between 80% and 20% of the initial intensity, barring exceptions set out in the methods). (b–e) Distributions of induction stage duration for different concentrations of CuCl_2_. When the induction phase duration *t* was less than the time t* between adding the reagent and starting the recording, the data is recorded as *t* ≤ t*. (f–i) Distributions of reaction gradient for different concentrations of CuCl_2_. The horizontal scale in (f) is larger than in (g–i).

The intensity-time series of single faceted spheroidal Mg NPs reacting with CuCl_2_ (0.1–7.4 mM) also displayed an induction stage (up to ∼260 s) followed by a decrease in scattering signal indicative of galvanic replacement (Fig. 6a). The induction-reaction behaviour was also apparent in spectrally resolved intensity time series (Fig. 4c and S10). Unlike the reaction with Na_2_PdCl_4_, the induction and reaction timescales were of the same order of magnitude, allowing the reaction stage to be resolved and analysed. Linear models were fitted to the reaction interval to use the intensity-time gradients as a proxy for the reaction rate; *R*-squared values >0.9 (Fig. S5) indicate that linear fits were suitable.

Increasing the concentration of CuCl_2_ shortened the induction stage duration (Fig. 6b–e). In some intensity time traces, no induction stage was observed after the start of the recording at ∼30 s (*t* ≤ t* in Fig. 6); their proportion increased with CuCl_2_ concentration (Fig. 6b–e), suggesting again shorter induction stages for larger concentrations. Distributions of initial scattering intensities (a proxy for initial NP size) do not vary systematically with CuCl_2_ concentration (Fig. S6), and initial intensities and induction stage durations are not correlated (Fig. S16).

Meanwhile, the reaction gradient increased with CuCl_2_ concentration, indicating that the rate of the galvanic replacement correlated with Cu^2+^ concentration. The induction stage durations for CuCl_2_ are shorter than those of Na_2_PdCl_4_ and Na_2_PtCl_4_, but longer than that of HAuCl_4_. This is consistent with the pH trend of the salts, however the different shape used for Cu reactions (spheroids) and Pd, Pt, and Au (platelets) cannot be ruled out at this point to play a role in the reaction kinetics.

### Designing galvanic replacement reactions

**Fig. 7:**
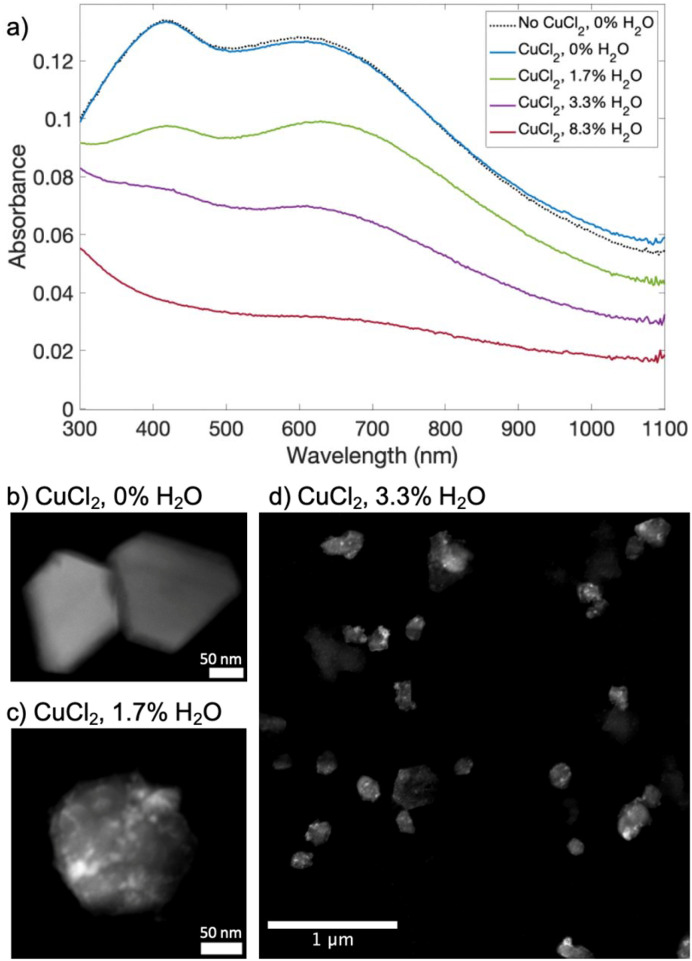
Water-assisted galvanic replacement. (a) UV-Visible absorbance spectra of faceted spheroidal Mg NPs redispersed in isopropanol after 10 minutes of reaction with (and without) CuCl_2_ and various concentrations of water. (b–d) HAADF-STEM images of NPs after reaction, where Cu islands can only be seen in reactions involving water. Additional images reported in Fig. S18.

The mechanistic insights provided by single particle investigations can inform the design of bulk colloidal reactions. We conducted partial galvanic replacement reactions of colloidal faceted spheroidal Mg NPs with CuCl_2_ in ethanol (with a 2 : 1.7 Mg : Cu ratio) in the presence of up to 8.3 vol% water. Whereas no spectral changes occurred for particles kept in anhydrous ethanol or exposed to CuCl_2_ in anhydrous ethanol after 10 minutes, the addition of water enabled galvanic replacement, as supported by the absorbance decay indicative of Mg oxidation (Fig. 7). Control experiments with no CuCl_2_ and the same water concentrations show no appreciable change in UV-Visible absorbance spectra over the same reaction time (Fig. S17), ruling out a direct reaction with water. Further, clear evidence of Cu clusters on Mg for reactions with CuCl_2_ in the presence of water contrasts with the smooth, uniform, and Cu-free NPs observed in anhydrous reactions (HAADF-STEM in Fig. 7, additional HAADF-STEM and SEM in Fig. S18), again indicating the enabling role of water in galvanic replacement of Mg by Cu. These results, using single particle insights, pave the way for further bulk reaction design utilising water as an initiator.

## Conclusions

The evolution of the scattering response of single Mg NPs during reaction with water (corrosion) and galvanic replacement by Pd, Cu, Pt, and Au was investigated. By systematically varying the concentrations of reactive species, observing reaction dynamics at the single particle level on a statistically relevant number of particles, and performing validating numerical calculations, we unravelled reaction processes otherwise invisible by bulk, ensemble-averaged approaches. Namely, corrosion starts immediately and occurs gradually, at a rate dependent on the water concentration. Meanwhile, in galvanic replacement by Pd, Cu, and Pt, we observed an induction stage before the start of the reaction. This induction stage lasted up to two hours and depended on the metal ion concentration and acidity. Significant shortening of the induction stage was observed when adding water or NaCl, pointing to the need for hydration and breakdown of the initially protective MgO layer for the reaction to proceed.

The knowledge gained from single particle studies, in particular the effect of water on the galvanic replacement of Mg NPs, was utilised to design the bulk reaction of Mg NPs with CuCl_2_. Water addition enabled the creation of bimetallic structures without significantly affecting the optical properties bestowed by the underlying Mg.

Together, these mechanistic insights into the breakdown of the MgO layer and the galvanic replacement of Mg provide tools to design synthetic strategies for bimetallic nanostructures, where the core Mg can be retained for, *e.g.*, its plasmonic properties and the decorating metals can be controlled in size and morphology to dictate their function, for instance as sensors or catalytic surfaces.

## Author contributions

A. L. Y. Brabant: data curation, formal analysis, investigation, methodology, validation, visualisation, writing – original draft, writing – review & editing. P. J. Knight: conceptualisation, formal analysis, investigation, methodology, validation, visualisation, writing – review & editing. K. Joyce: conceptualisation, formal analysis, investigation, methodology, validation, visualisation, writing – review & editing. M. Elabbadi: conceptualisation, formal analysis, investigation, methodology, visualisation, writing – review & editing. C. Boukouvala: formal analysis, validation, investigation, methodology, software, writing – review & editing. Vladimir Lomonosov: investigation, methodology, resources, writing – review & editing. Emilie Ringe: conceptualisation, funding acquisition, project administration, resources, supervision, writing – original draft, writing – review & editing.

## Conflicts of interest

There are no conflicts of interest to declare.

## Supplementary Material

NR-018-D6NR00806B-s001

## Data Availability

The processed data and Python/MATLAB scripts required to produce the figures in the manuscript, as well as Fig. S1–S6, S10d, S12, and S14–S17 in the SI, are available in Cambridge University's Apollo repository at https://doi.org/10.17863/CAM.127693. Raw hyperspectral data is available upon request. The code for DDSCAT can be found at https://ddscat.wikidot.com with https://doi.org/10.1364/JOSAA.11.001491. The version of the code employed for this study is version 7.3.2. Supplementary information (SI): additional experimental and statistical analysis details and results, Mg nanoparticle size distributions from SEM, UV-Vis-NIR spectra, SEM and HAADF-STEM images, SEM-EDS spectra, single particle optical scattering spectral time series, photographs and spectra taken in the dark field optical microscope, results of additional numerical calculations, control experiments, and results for the galvanic replacement of Mg nanoparticles by HAuCl_4_ and Na_2_PtCl_4_. See DOI: https://doi.org/10.1039/d6nr00806b.
