# Supplementary material for: The reactivity of single magnesium nanoparticles towards corrosion and galvanic replacement
Source: Nanoscale. 2026 Jun 2;18(27):14310–21. doi: 10.1039/d6nr00806b (PMC13267779; doi:10.1039/d6nr00806b)
Supplement: NR-018-D6NR00806B-s001 [file NR-018-D6NR00806B-s001.pdf]

Supplementary Information (SI) for Nanoscale.
This journal is © The Royal Society of Chemistry 2026

Supplementary Information for

The reactivity of single magnesium nanoparticles towards corrosion and galvanic replacement†

Ambre L.Y. Brabant,[a] *Pip J. Knight,*[b] *Katharine M. Joyce,*[b] *Mohsen Elabbadi,*[b] *Vladimir Lomonosov,*[b] *Christina Boukouvala,*[b] *Emilie Ringe*[a,b*]

a. Department of Earth Sciences, University of Cambridge, Downing Street, Cambridge CB2 3EQ, United Kingdom

b. Department of Materials Science and Metallurgy, University of Cambridge, 27 Charles Babbage Road, Cambridge CB3 0FS, United Kingdom

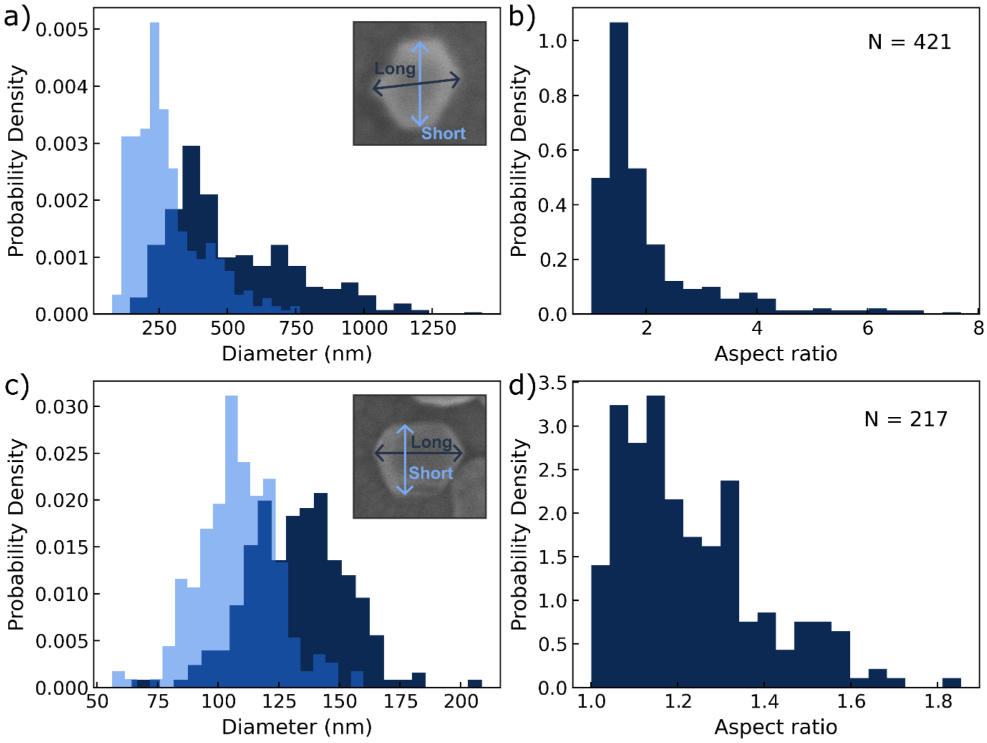

Figure S1. Size distribution of (a) platelet Mg NPs and (c) faceted spheroidal Mg NPs obtained from SEM images. Dark and light blue histograms represent the length along the long and short axis, respectively, as illustrated in the inset. b) Distribution of aspect ratios (long axis divided by short axis) of (b) platelet Mg NPs and (d) faceted spheroidal Mg NPs. *N* indicates the number of NPs measured.

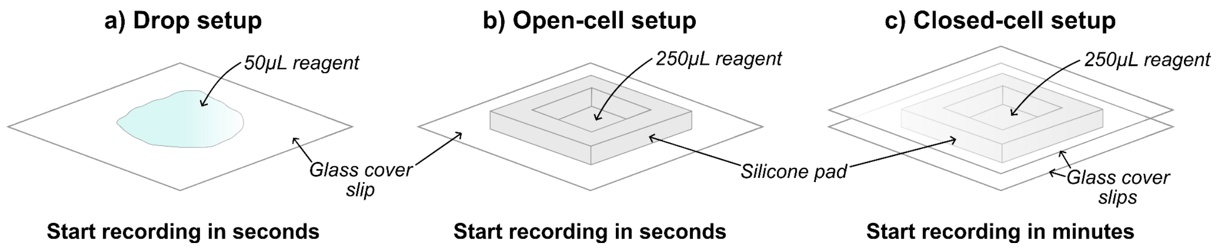

Figure S2. The three experimental setups described in the methods.

Table S1. Proportions of single particle intensity-time series in galvanic replacement experiments with more than one step. For $CuCl_2$ reactions, fewer non-single step intensity-time series are observed for larger $CuCl_2$ concentrations. This is possibly a trend, although it is not observed in Na_2PdCl_4 traces; the trend could be attributed to a smaller proportion of aggregates on the coverslip, or a faster reaction leading to the occurrence of a first step before the start of experimental recording.

Experiment	Percentage of >1 step single particle intensity-time series
2 mM Na_2PdCl_4	34
1.5 mM Na_2PdCl_4	16
1.0 mM Na_2PdCl_4	27
0.5 mM Na_2PdCl_4	42
1.5 mM Na_2PdCl_4+ 1 vol.% H_2O	39
0.5 mM Na_2PdCl_4+ 2 mM NaCl	44
7.4 mM $CuCl_2$	17
1.0 mM $CuCl_2$	23
0.5 mM $CuCl_2$	34
0.1 mM $CuCl_2$	56

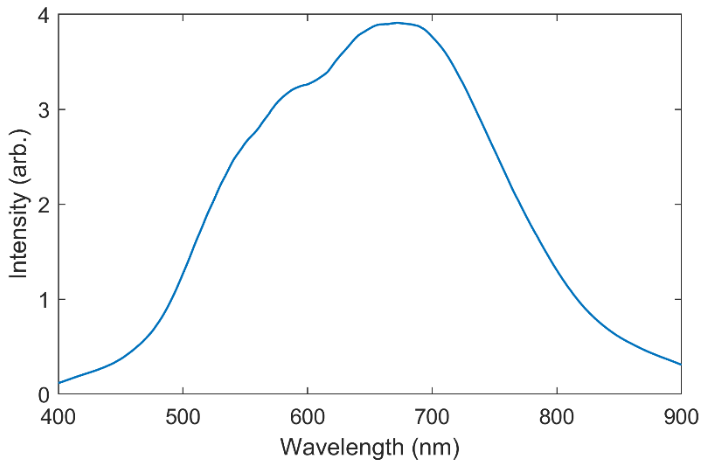

Figure S3. Spectrum of the halogen lamp used in the optical microscope setup.

Table S2. Surface energies used in a Wulff construction to approximate the shape of the faceted spheroidal Mg NPs. Only the relative magnitudes of the values are relevant as they determine the shape of the particle. Values from ref.[1]

Facet	Surface Energy (meV Å^{-2})
$\{0001\}$	34.6
$\{10\bar{1}0\}$	39.9
$\{10\bar{1}1\}$	40.9
$\{11\bar{2}1\}$	45.7

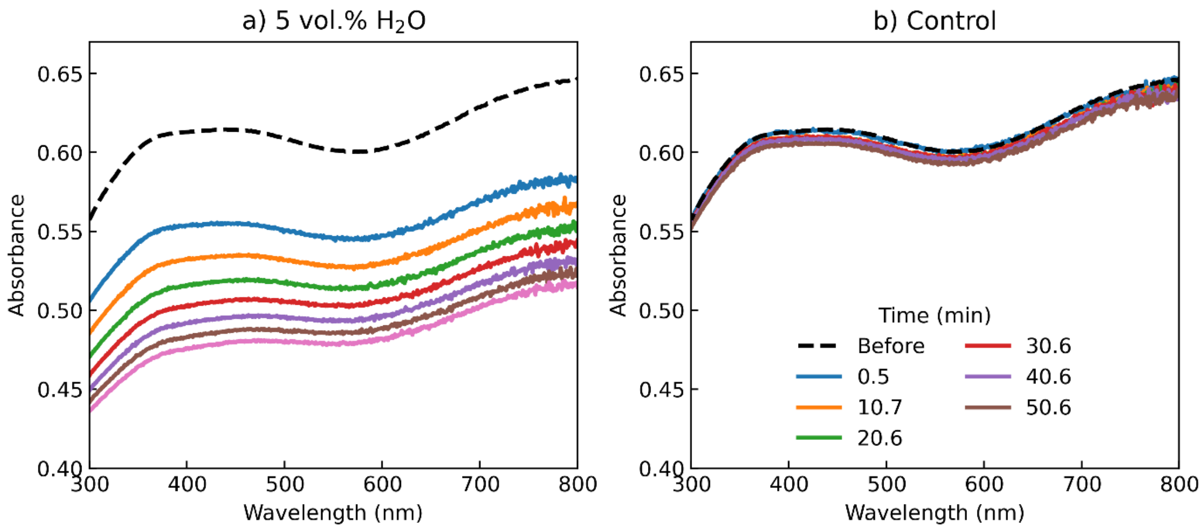

Figure S4. Bulk UV-Visible absorbance spectrum of a suspension of faceted spheroidal Mg NPs in a) a 5 vol.% water in isopropanol mixture, showing corrosion, and b) dry isopropanol, showing only a small decrease indicative of slow sedimentation. In a), the dashed black line is a spectrum in isopropanol with a concentration adjusted to match the dilution once the water is added. Data acquired on a Thermo-Scientific Evolution 220 UV-visible spectrophotometer for a ~0.2 µM suspension of faceted spheroidal Mg NPs.

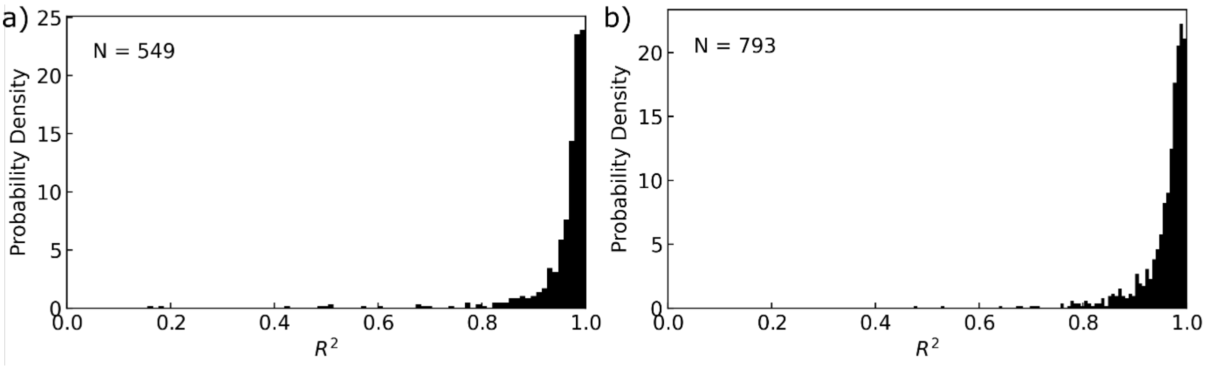

Figure S5. Distributions of R-squared values associated with linear fits to intervals of scattering intensity-time series. a) Fits between 100% and 5% of the initial intensity in intensity-time series of the corrosion reaction with water-isopropanol mixtures. b) Fits between 80% and 20% (sometimes 50%, see methods in the main text) of the initial intensity in intensity-time series of the galvanic replacement reaction with $CuCl_2$. *N* indicates the number of values in the distributions.

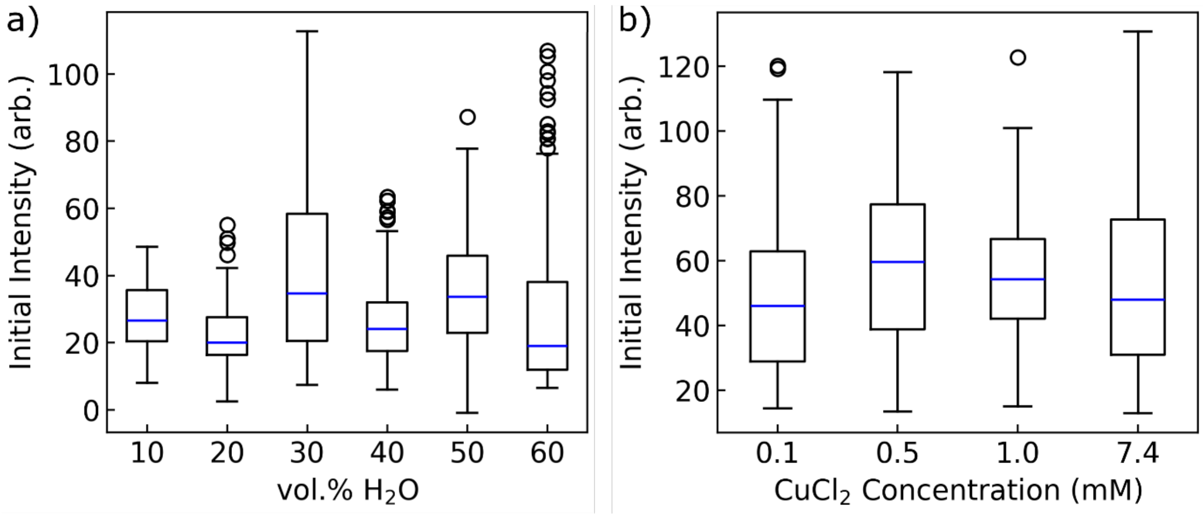

Figure S6. Lack of systematic correlation between initial scattering intensity and concentration. Box-and-whisker plots giving the distributions of single particle initial scattering intensities in the colour camera for a) corrosion experiments and b) galvanic replacement experiments with $CuCl_2$. Blue lines give the median, and boxes correspond to the interquartile range between the first and third quartiles. Whiskers indicate the farthest point within 1.5 times the interquartile range of the box. Circles indicate data points outside of the whiskers.

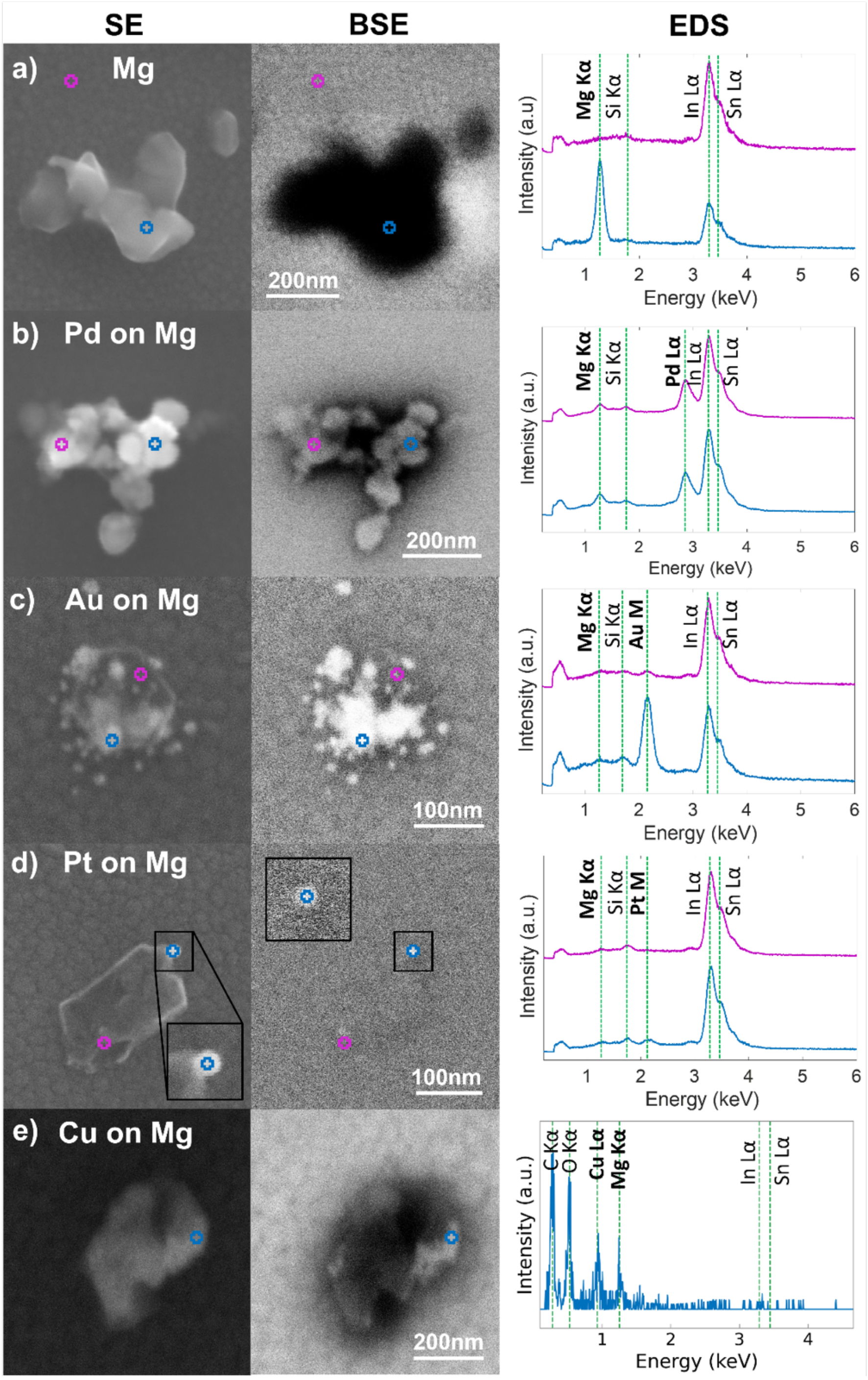

Figure S7. Products of partial galvanic replacement, imaged by a secondary electron detector (SE), backscattered electron detector (BSE), and analysed by energy-dispersive X-ray spectroscopy (EDS). Corresponding SE and BSE images have the same field of view. Color-coded markers indicate where EDS spectra were acquired, and insets in d) show a magnified region. Mg NPs a) not exposed to a reagent, and after partial reactions with b) Na_2PdCl_4, c) $HAuCl_4$, d) Na_2PtCl_4, and e) $CuCl_2$. Mg NPs were drop-cast onto indium-doped tin oxide (ITO)-coated cover slips and dried. 250 µL (50 µL for $CuCl_2$) of metal salt solution was added and allowed to react for a fixed length of time appropriate to reach partial replacement before quenching by rinsing with isopropanol. Platelet Mg NPs were reacted with 1.5 mM Na_2PdCl_4 for 7.5 min, 0.05 mM $HAuCl_4$ for 15 s, or 1.0 mM Na_2PtCl_4 for 60s. Faceted spheroidal Mg NPs were reacted with 0.1 mM $CuCl_2$ for 2 min. $CuCl_2$ products were imaged at 10 kV, others at 15 kV.

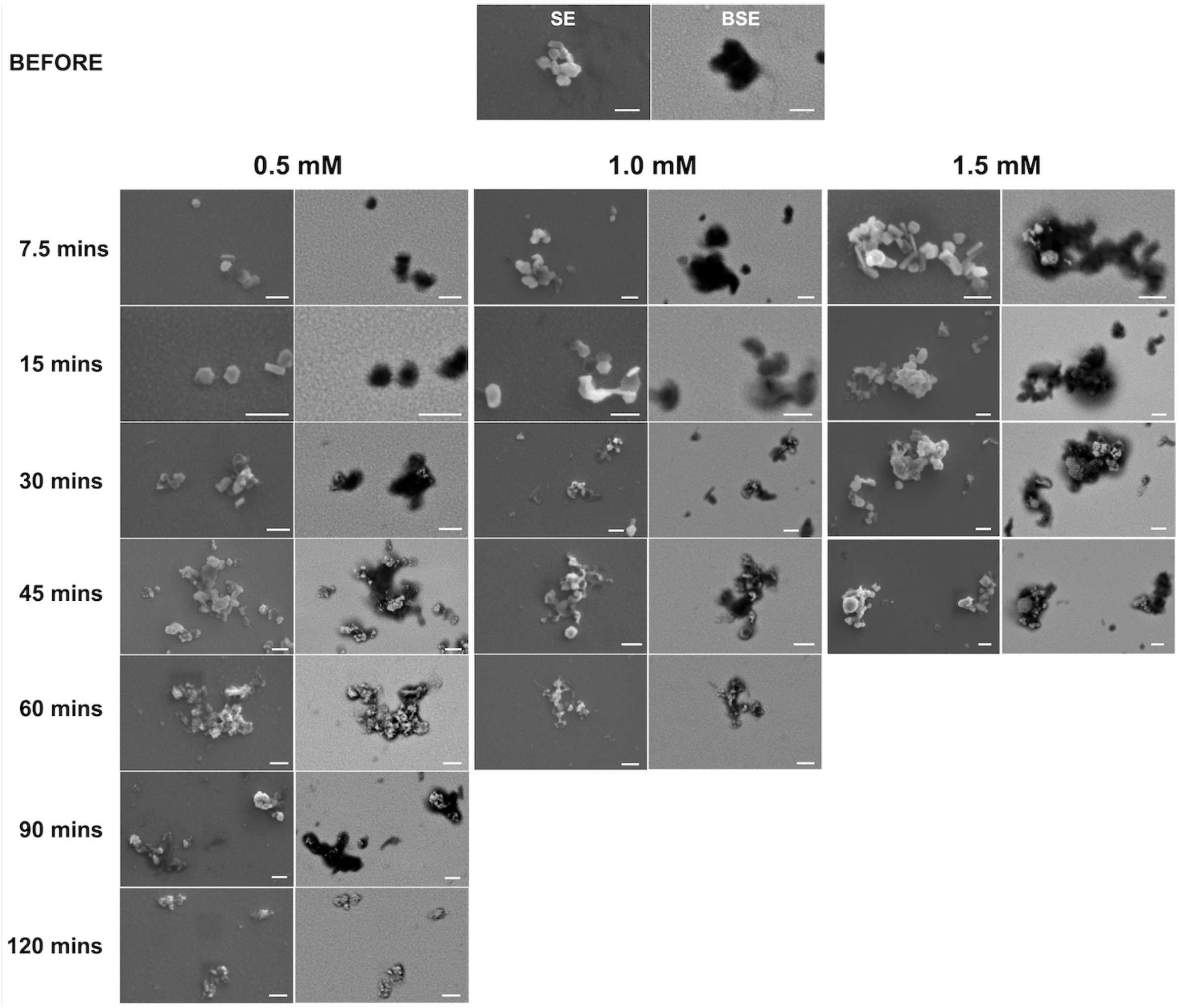

Figure S8. SE and BSE images of the products of reactions between platelet Mg NPs immobilised on ITO-coated coverslips and various concentrations of Na_2PdCl_4 in isopropanol, carried out as described in Figure S7. The top row shows Mg NPs not exposed to Na_2PdCl_4, and subsequent rows show products of reactions quenched at different times. Scale bars, 400 nm.

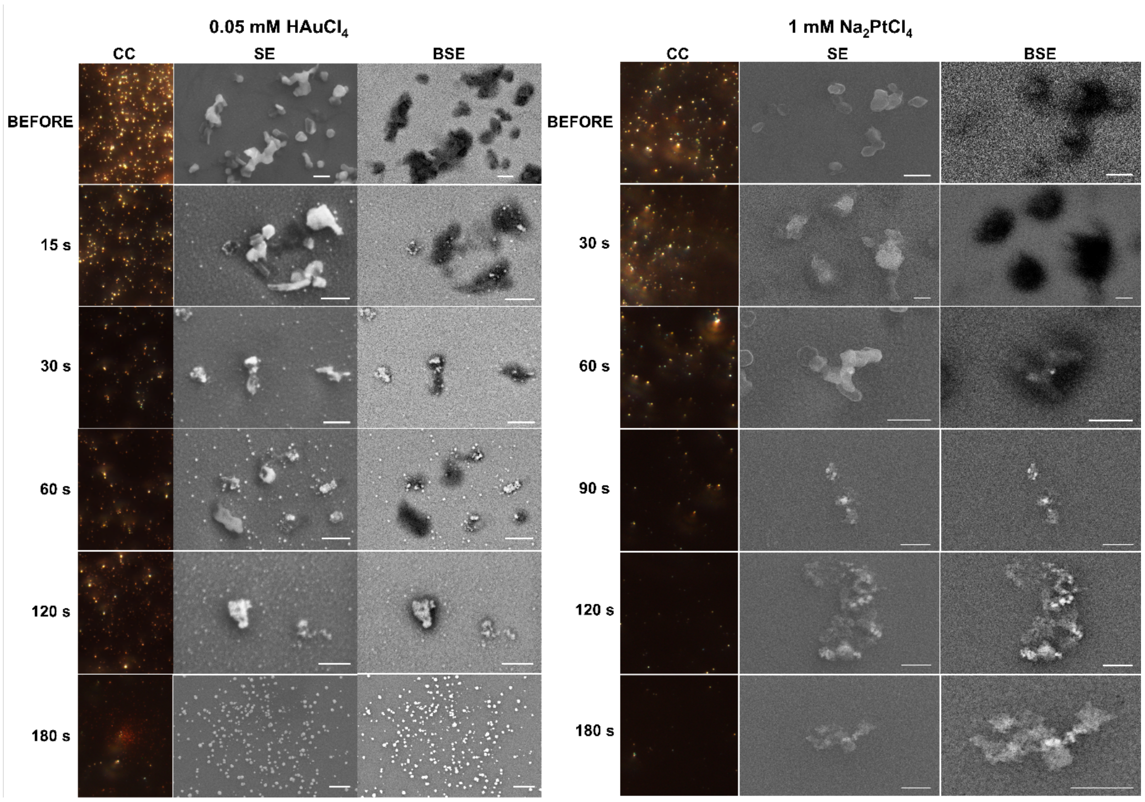

Figure S9. Colour camera (CC), SE, and BSE images of the products of reactions between platelet Mg NPs immobilised on ITO-coated coverslips and 0.05 mM $HAuCl_4$ or 1 mM Na_2PtCl_4 in isopropanol, carried out as described in Figure S7. The top row shows Mg NPs not exposed to reactants, and subsequent rows show products of reactions quenched at different times, alongside the CC images showing reaction progress via dimming of the scattering signal. Scale bars, 400 nm. CC images have a width of ~30 µm.

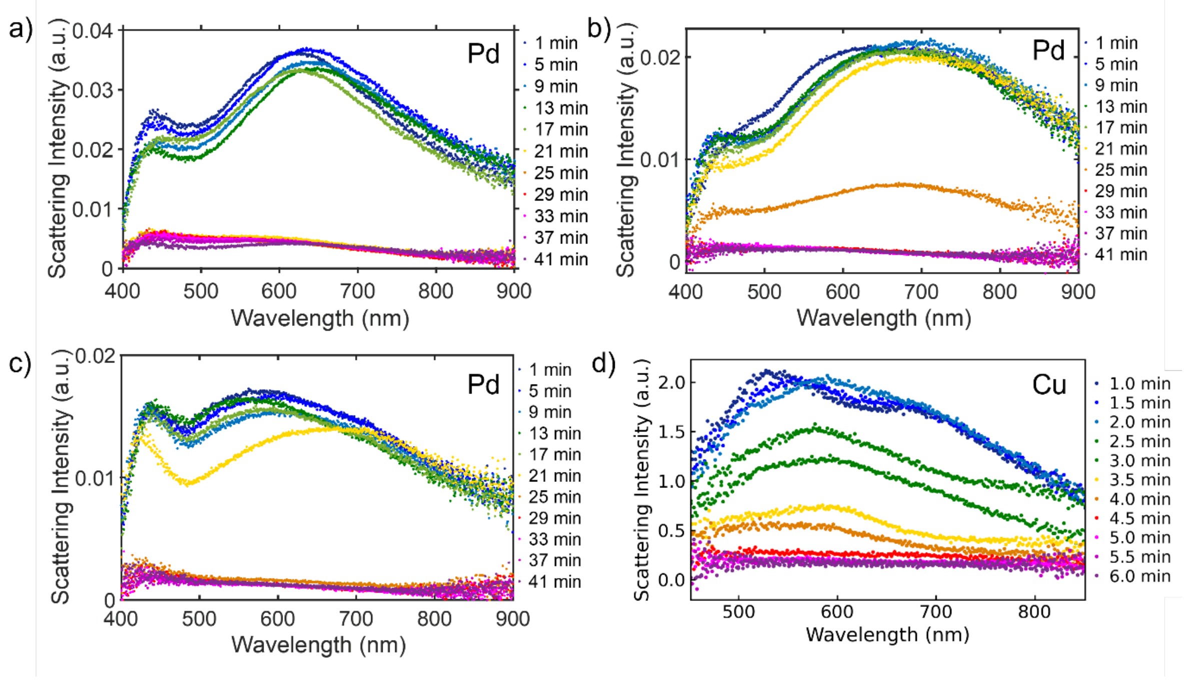

Figure S10. Spectral time series of single NPs reacting with a-c) 1.0 mM Na_2PdCl_4 and d) 0.1 mM $CuCl_2$.

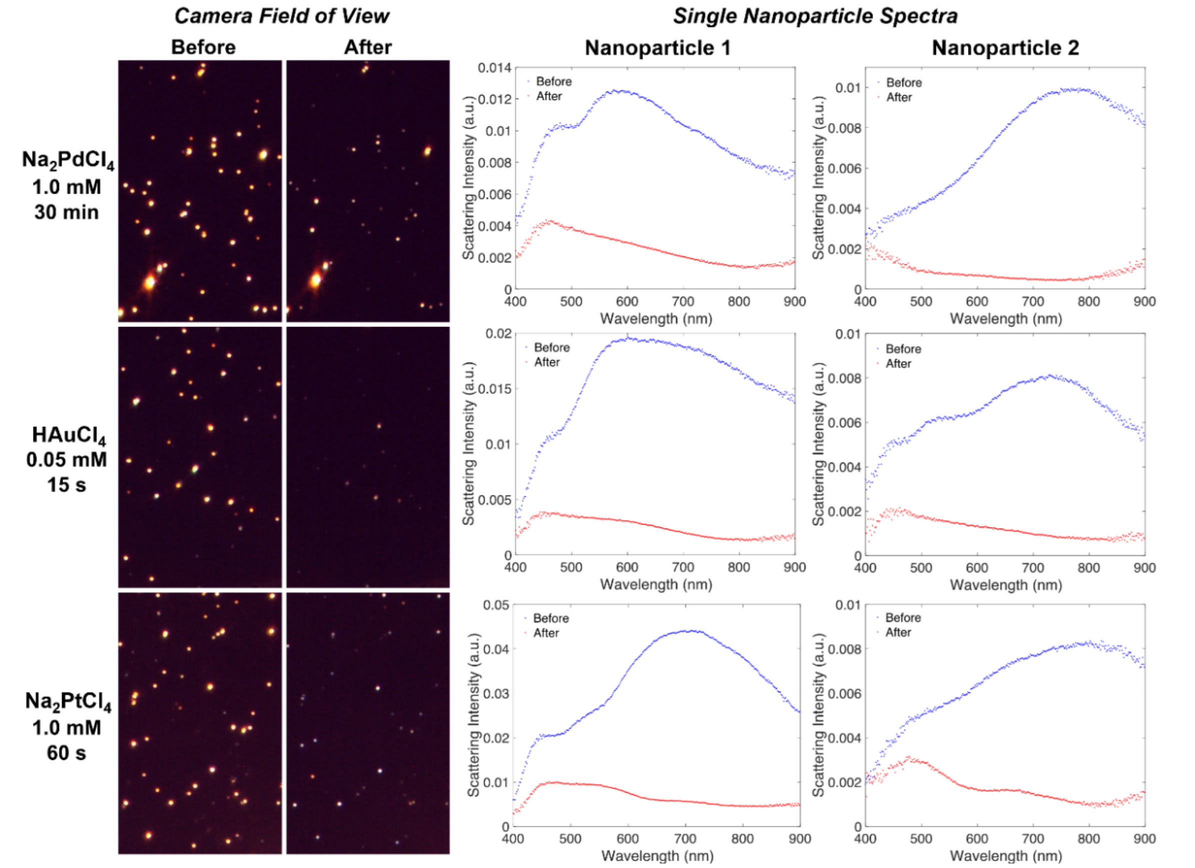

Figure S11. Snapshots of the CC and single NP spectra measured before and after galvanic replacement of platelet Mg NPs by Na_2PdCl_4, $HAuCl_4$, or Na_2PtCl_4. Camera images have a width of ~30 µm. Prior to adding the metal salt solution, the region of interest shown in the camera image was scanned using a piezoelectric stage and a Princeton Instruments IsoPlane SCT320 spectrometer fitted with a 50g/mm grating followed by a Princeton Instruments ProEM HS 1024 × 1024 EMCCD. After the reaction, the slide was rinsed with isopropanol and allowed to dry before acquiring another hyperspectral map. The resulting hyperspectral maps were corrected for the lamp profile and background.[2] Single particle spectra were obtained by averaging a 3 by 4 pixels region (corresponding to a 0.90 µm by 0.56 µm area) around the NP centre.

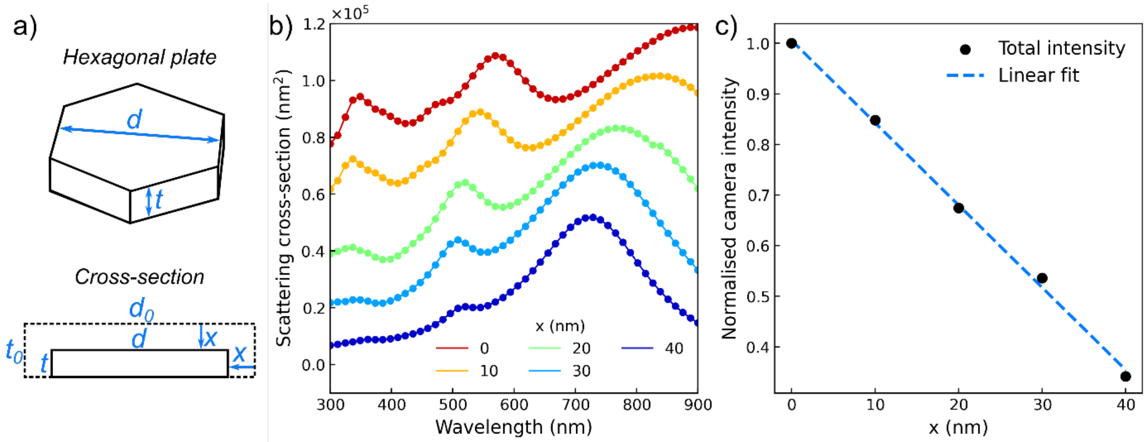

Figure S12. Calculated scattering by platelet Mg NPs, modelled as described in the methods except for an interdipole distance of 1.5 nm, with light incoming to the substrate at 39˚, a homogeneous surrounding dielectric environment of refractive index 1.33 (water), and no glass substrate. a) Schematic of the hexagonal platelet used in the simulations; the face-to-face diameter d and a thickness t have initial values t_0 = 52 nm and d_0 = 225 nm. The upper and lateral crystal faces each retreat by x, while the bottom face does not retreat as it is in contact with the substrate. b) Calculated scattering cross-sections as a function of x. c) Calculated intensity in the camera as a function of x. Markers in b) and c) indicate calculated values.

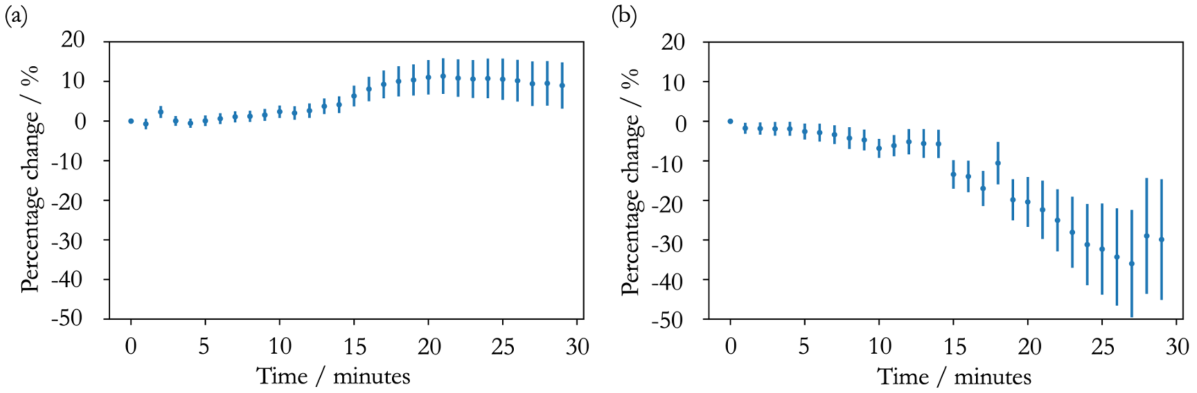

Figure S13. Average intensity change for NPs in the camera field of view as a function of time (error bars are standard deviation), for NPs immersed in a) isopropanol, and b) 1 vol.% water in isopropanol performed in a closed-cell setup. In a), the intensity increased slightly, likely because of defocussing over time. In b), the average intensity decreased by nearly 40% in 30 minutes.

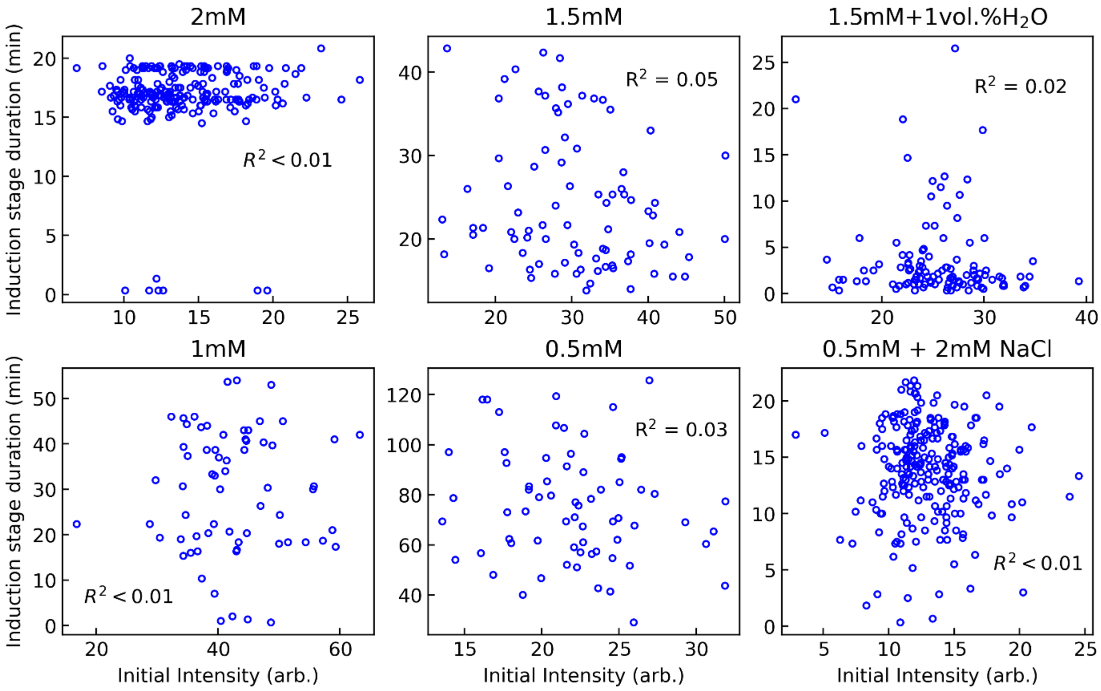

Figure S14. Induction stage duration against initial intensity for single particle reactions with Na_2PdCl_4, showing no correlation between the two variables. Plot titles give the concentration of Na_2PdCl_4. In addition to NP size, aggregation and optical alignment could also influence the absolute initial intensity.

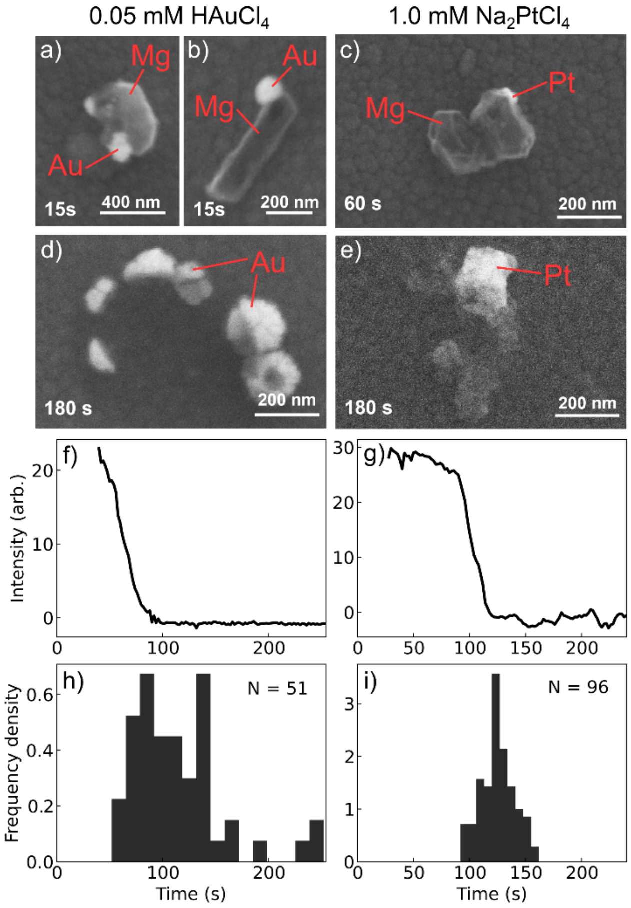

Figure S15. Reaction of platelet Mg NPs with $HAuCl_4$ and Na_2PtCl_4. a-e) SE images of products formed by reactions of various durations. a-c) products of partial reactions, consisting of Au or Pt decorations on Mg NPs. d-e) products of complete reactions, consisting of clusters of Au or Pt NPs having completely replaced Mg. Single particle intensity-time series examples in a reaction with f) 0.05 mM $HAuCl_4$ and g) 1.0 mM Na_2PtCl_4. Distribution of single particle decay times (defined as the time for the intensity to drop to 15% of its initial value) in a reaction with h) 0.05 mM $HAuCl_4$ and i) 1.0 mM Na_2PtCl_4.

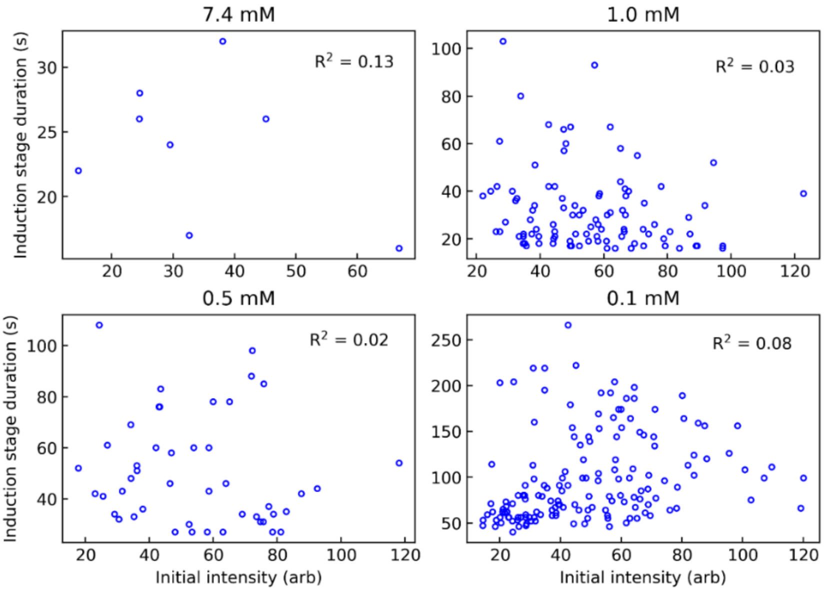

Figure S16. Induction stage duration against initial intensity for single particle reactions with $CuCl_2$, showing no correlation between the two variables. Plot titles give the concentration of $CuCl_2$. In addition to NP size, aggregation and optical alignment could also influence the absolute initial intensity.

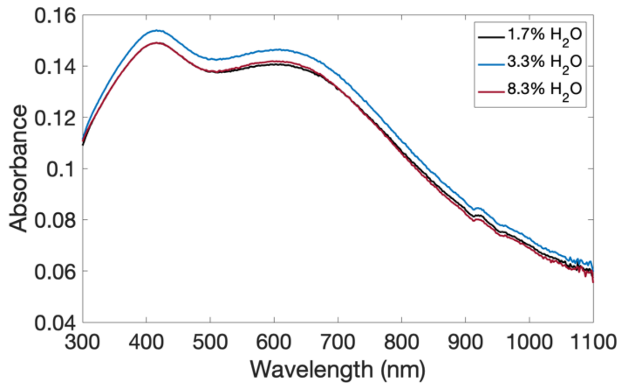

Figure S17. UV-Visible absorbance spectra of colloidal suspensions of faceted spheroidal Mg NPs exposed to various concentrations of water for 10 minutes, then centrifuged and redispersed in ethanol.

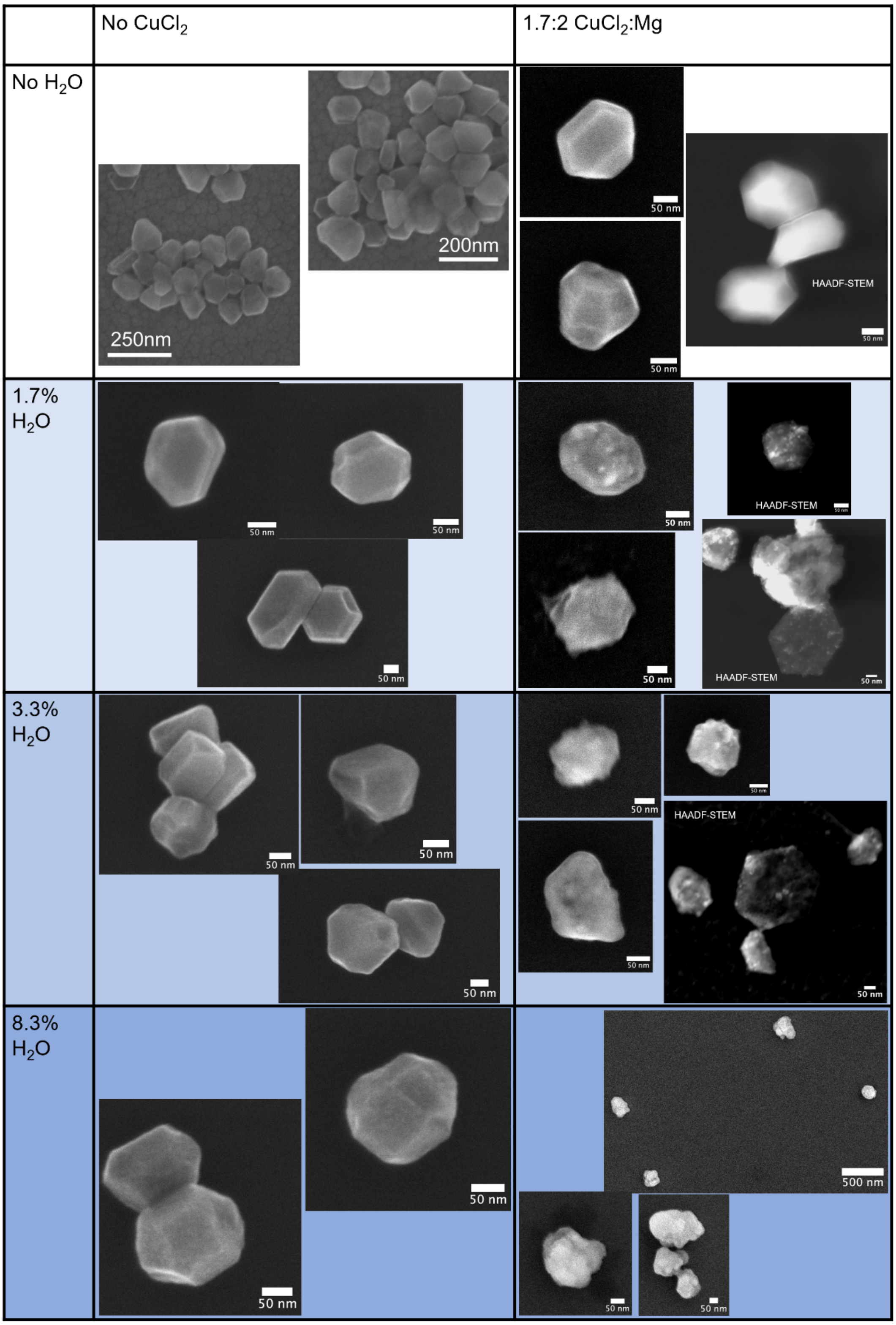

Figure S18. Electron micrographs of faceted spheroidal Mg NPs after 10 minutes of exposure to various concentrations of H_2O and $CuCl_2$ in ethanol. Micrographs are SEM (no label) and HAADF-STEM images.

References

1. Kopač Lautar, A.; Kopač, D.; Rejec, T.; Bančič, T.; Dominko, R. Morphology Evolution of Magnesium Facets: DFT and KMC Simulations. *Physical Chemistry Chemical Physics* **2019**, *21* (5), 2434–2442. https://doi.org/10.1039/c8cp06171h.
2. Kumar, A.; Villarreal, E.; Zhang, X.; Ringe, E. Micro-Extinction Spectroscopy (MExS): A Versatile Optical Characterization Technique. *Advanced Structural and Chemical Imaging* **2018**, *4* (1), 8. https://doi.org/10.1186/s40679-018-0057-6.
